# Psychosocial Factors Associated With Medication Burden Among Patients With Type 2 Diabetes Mellitus: A Cross-Sectional Study

**DOI:** 10.1155/jdr/8885209

**Published:** 2025-05-29

**Authors:** Minh Hoang Le, Ngoc Diem Le, Thi Thuy Nhu Le, Minh Cuong Nguyen, Van De Tran

**Affiliations:** ^1^Faculty of Traditional Medicine, Can Tho University of Medicine and Pharmacy, Can Tho, Vietnam; ^2^General Hospital of O Mon District, Can Tho, Vietnam; ^3^Faculty of Pharmacy, Nam Can Tho University, Can Tho, Vietnam; ^4^Department of Health Organization and Management, Can Tho University of Medicine and Pharmacy, Can Tho, Vietnam

**Keywords:** depression, medication beliefs, medication burden, psychosocial factors, self-efficacy, Vietnam, with Type 2 diabetes

## Abstract

**Background:** Previous research has focused largely on sociodemographic, clinical, and medication-use characteristics, overlooking the critical role psychosocial factors may play in influencing the medication-related burden among patients with Type 2 diabetes mellitus.

**Objectives:** The aim of this study was to explore the psychosocial factors associated with medication burden among patients with Type 2 diabetes mellitus.

**Methods:** A cross-sectional study was performed on 261 patients with Type 2 diabetes from four community health stations in Can Tho, Vietnam. Linear regression models were used to identify demographic and health factors, as well as psychosocial factors, including medication self-efficacy, medication social support, satisfaction with medication treatments, depression, and medication beliefs that are related to medication burden.

**Results:** The presence of dyslipidemia (*β* = 0.15, *p* = 0.002) and depression (*β* = 0.21, *p* < 0.001) and a stronger belief in the necessity of medication (*β* = 0.41, *p* < 0.001) were significantly associated with increased medication-related burden. Conversely, higher education (*β* = −0.16, *p* = 0.002), a greater number of family members (*β* = −0.25, *p* < 0.001), and higher medication self-efficacy (*β* = −0.21, *p* < 0.001) were significantly associated with a reduction in medication-related burden.

**Conclusion:** Psychosocial factors were found to be associated with medication burden among patients with Type 2 diabetes. These findings highlight the importance of psychosocial factors in managing medication burden. Future interventions should focus on these factors to reduce the medication burden and improve the quality of life for patients with Type 2 diabetes.

## 1. Introduction

Diabetes is a major contributor to increasing morbidity and mortality rates, significantly impacting global health [1]. In 2021, an estimated 537 million adults aged 20–79 years worldwide were living with diabetes, representing approximately 10.5% of the global adult population [2]. Of these, nearly half were unaware of their condition [2]. By 2045, the number of people with diabetes is projected to rise to 783 million, a nearly 46% increase from 2021 [2]. In Vietnam, diabetes also imposes a substantial disease burden, with an estimated 5.2 million adults aged 20–79 years living with the condition in 2021, accounting for approximately 6.8% of the adult population [3]. By 2045, the number of people with diabetes in Vietnam is expected to reach 7.4 million, a 42.3% increase from 2021 [3]. This rapid increase places Vietnam among countries with a notable increase in diabetes prevalence [4]. However, only 28.9% of people with diabetes are managed at medical facilities [5].

The Vietnamese healthcare system is organized into four levels, from central to commune levels, where the central and provincial levels provide tertiary and secondary care with specialized healthcare professionals, while the district and commune levels handle primary care [6]. Despite national strategies in healthcare for diabetes, care remains largely hospital- and specialist-centric [7], with only 53% of commune health centers providing diabetes treatment services and 3% having at least one type of diabetes medication [8]. A study in Vietnam found that 17.0% of elderly outpatients with diabetes forgot to take their medication [9]. This rate was even higher in another study by Nguyen et al., showing that 24% of elderly patients with Type 2 diabetes did not adhere to their medication regimen [10]. Drug-related problems are common during the prescription stage and are associated with patient medication behaviors, with an average of 1.54 ± 1.07 drug-related problems per prescription [11]. Among these, adverse drug reactions are the most common, accounting for 68.8% [11].

The treatment of diabetes, a chronic noncommunicable disease, is lifelong and typically involves the use of multiple medications to achieve glycemic control [12]. In the United States, adults with diabetes use an average of 5.9 prescription medications with a standard error of 0.11 [13]. In some Asian countries, the average is 6.7 medications with a standard deviation (SD) of 2.5 [14]. A study in Vietnam revealed that diabetes outpatients used an average of 3.92 medications with a SD of 1.28 [11]. The prolonged use of several medications can pose challenges and become burdensome for patients [12]. While this use of multiple medications is necessary for effective treatment, it can lead to problems such as side effects, drug interactions, and overall medication load [15], making daily life more difficult for patients in terms of medication adherence, management, and travel [12], thereby increasing the burden on patients [15].

The medication-related burden (MRB) is a component of the overall treatment burden, reflecting the negative experiences patients encounter during their medication treatment [16]. Mohammed et al. outlined five key dimensions of MRB: (1) the strain of medication routines, (2) challenges related to medication characteristics, (3) adverse effect burdens, (4) issues connected to healthcare and medication management, and (5) social impacts associated with medication use [17]. MRB can contribute to nonadherence and poor clinical outcomes and impact patient satisfaction, psychological well-being, social functioning, and overall quality of life [16].

Previous studies on patients with diabetes have shown that the medication burden is influenced by various factors. These include demographic factors such as male sex, age over 60 years, marital status, and higher educational attainment [18]; clinical characteristics such as uncontrolled diastolic blood pressure, elevated blood glucose levels, high HbA1c, emergency department admissions, and fasting blood glucose levels [12, 16, 18]; complications such as neuropathy or retinopathy [12]; and other factors such as pain intensity and medication adherence [16]. However, the role of psychosocial factors in affecting MRB among patients with diabetes remains underexplored, highlighting a significant gap in current understanding and an area that warrants further investigation. The role of psychosocial factors in management and treatment is now widely accepted, and, as a result, medicine has shifted from a purely biological perspective on causation and management to a more biopsychosocial model of disease and care [19].

Both individual and multidimensional psychosocial issues reduce patients' ability to fully participate in treatment, leading to an increased treatment burden [20]. Psychosocial factors such as satisfaction with medication treatments, medication self-efficacy, and depression have been shown to be associated with medication burden, particularly in older adults with multimorbidity [21]. These findings suggest that similar psychosocial factors may also influence the medication burden in patients with diabetes. A better understanding of these psychosocial determinants of medication burden could help alleviate patients' perceived burden, improve self-management, and enhance treatment adherence. Furthermore, to the best of our knowledge, no studies have been conducted on medication burden in chronic patients in Vietnam. Therefore, this study was conducted to explore the psychosocial factors associated with medication burden among patients with Type 2 diabetes in Vietnam.

## 2. Method

### 2.1. Study Design

A descriptive cross-sectional study was conducted on patients with Type 2 diabetes recruited from community health stations in Can Tho, Vietnam. Can Tho City is divided into eight administrative units, including four urban districts and four rural districts. For this study, two urban districts (Ninh Kieu and O Mon) and two rural districts (Co Do and Phong Dien) were randomly selected. From the list of all commune health stations within these districts, one health station in each district was randomly chosen to participate in the study. These community health stations, which provide primary healthcare services, play crucial roles in managing, diagnosing, and treating patients with Type 2 diabetes in the local population.

### 2.2. Participants

The study participants were outpatients diagnosed with Type 2 diabetes, which was confirmed through physician assessment and patient medical records. The eligibility criteria included individuals aged 18 years and older who had been prescribed at least one medication for diabetes treatment. The exclusion criteria included patients diagnosed with gestational diabetes, those exhibiting signs of cognitive impairment, and individuals who did not provide consent to participate. Participants were consecutively recruited over a four-week period from July 15 to August 9, 2024. With the use of a convenience sampling method, the data collectors distributed self-administered questionnaires to patients while they waited for their medications at the health stations, which continued until 75 questionnaires were collected from each station.

### 2.3. Measurements

A printed, self-administered questionnaire in Vietnamese, consisting of three main sections, was used to collect data. The first section included general demographic information such as age (years), sex, education level, marital status, employment status, number of family members, and health characteristics such as number of prescribed medications, duration of diabetes (years), and comorbid conditions. The second section utilized scales to explore psychosocial factors, including medication self-efficacy, medication social support, satisfaction with medication treatments, depression, and beliefs about medication.

Medication self-efficacy was assessed using the Self-Efficacy for Appropriate Medication Use Scale (SEAMS), a 13-item tool designed to evaluate patients' confidence in using their medications correctly [22]. Each item was rated on a scale from 1 (*unconfident*) to 3 (*extremely confident*). The total score for medication self-efficacy ranged from 13 to 39, with higher scores indicating greater confidence. The Cronbach's alpha value in this study was 0.83, indicating acceptable reliability.

Medication social support was measured using the Medication-Specific Social Support Scale (MSSS), an 8-item tool designed to assess the level of social support that patients received in relation to their medication use [23]. Each item was rated on a Likert scale from 0 (*never*) to 4 (*very often*). The medication social support score was the average of the items, with higher scores indicating greater social support. The Cronbach's alpha in this study was 0.77, reflecting acceptable reliability.

Satisfaction with medication treatments was measured using the abbreviated Treatment Satisfaction Questionnaire for Medication (TSQM-9), a 9-item tool designed to assess patient satisfaction with their medication [24]. Each item was rated on a Likert scale from 1 (*extremely dissatisfied*) to 7 (*extremely satisfied*). The satisfaction score was calculated by summing the item scores and then converting the total score into a 0–100 scale, with higher scores indicating greater satisfaction. The Cronbach's alpha in this study was 0.93, indicating acceptable reliability.

Depression was assessed using the Vietnamese version of the 4-item Patient Health Questionnaire (PHQ-4), a tool designed to screen for depressive symptoms and previously used in Vietnamese populations [25]. Each item was rated on a scale from 0 (*not at all*) to 3 (*nearly every day*). The depression score was calculated by summing the items, with total scores ranging from 0 to 12, with higher scores reflecting greater severity of depressive symptoms. The Rasch item reliability for this scale was 0.96, indicating strong reliability.

Medication beliefs were assessed using the Vietnamese version of the Beliefs about Medicines Questionnaire (BMQ-V), an 18-item tool designed to measure patients' beliefs about medications and validated for Vietnamese populations [26], which is divided into four subscales: necessity (5 items), concerns (5 items), overuse (4 items), and harm (4 items). Each item was rated on a Likert scale from 1 (*strongly disagree*) to 5 (*strongly agree*). Subscale scores were calculated by summing the items, with higher scores indicating stronger beliefs in that particular aspect. The Cronbach's alpha for the scale in this study was 0.86, indicating acceptable reliability.

In the final section related to outcome measurement, medication burden was assessed using the first four items of the Treatment Burden Questionnaire (TBQ), a 15-item scale that is used to evaluate perceived treatment burden [27]. These four items were previously used in another study to assess medication burden [21]. Each item was rated on an 11-point Likert scale from 0 (*not a problem*) to 10 (*big problem*), with higher scores indicating greater perceived burden. The medication burden score was calculated by summing the four items, with total scores ranging from 0 to 40, where higher scores reflected a greater burden. The Cronbach's alpha for the scale in this study was 0.84, indicating acceptable reliability.

The TRAPD method was employed to translate the SEAMS, MSSS, TSQM-9, and TBQ into Vietnamese [28]. Initially, an English lecturer specializing in medical terms and a public health researcher from a Vietnamese university translated the questionnaire. A panel of experts, including a language specialist and clinical physicians, reviewed this translation. The team harmonized some nonstandardized terms to align with the original concepts during the review. After pretesting the final version with 20 patients with Type 2 diabetes at a community health station in Can Tho, no changes were made as the questions proved to be clear and straightforward. Each step of the translation process was meticulously documented.

### 2.4. Data Analysis

Data analysis was conducted via SPSS Version 22.0. The Shapiro–Wilk test and a Q–Q plot were used to assess the normality of the continuous data. The descriptive statistics included frequencies and percentages for categorical variables, means and SDs for normally distributed continuous variables, and medians with interquartile ranges (IQRs) for nonnormally distributed continuous variables. The associations between medication burden scores and categorical demographic and health variables were assessed using the Mann–Whitney *U* test for two categories and the Kruskal–Wallis H test for three or more categories. Spearman's rho was used to examine correlations between medication burden scores and continuous demographic, health, and psychosocial variables.

Univariate and multivariate linear regression models using the enter method were applied to identify factors associated with medication burden. Independent variables, including demographic, health, and psychosocial factors, were included in the univariate analysis, but only statistically significant variables from this univariate analysis were included in the multivariate regression. To prevent overfitting, only chronic conditions with a prevalence rate above 30% in the study sample were included in the analysis [21]. Independent variables with very strong correlations (at least 0.8) with other independent variables [29] but weaker correlations with the dependent variable were excluded to avoid multicollinearity. A variance inflation factor (VIF) threshold above 5 indicated multicollinearity in the regression analysis. All continuous variables in this study, except for age, had asymmetrical distributions and were transformed using the percentile-to-normal method prior to statistical inference [30]. All the results of all the statistical analyses were considered significant at the prespecified alpha level of 0.05.

## 3. Results

A total of 300 questionnaires were distributed, with 39 excluded due to incomplete responses (fewer than half of the questions answered), leaving 261 participants included in the analysis (response rate: 87%). Compared with males (36.0%) and those under 60 years of age (36.4%), females (64.0%) and individuals aged 60 years and older (63.6%) were more represented. Over 40% of the participants had an education level of lower secondary or below. The majority of participants were married (72.6%) and had comorbid hypertension (89.0%), as presented in Table 1. The median duration of Type 2 diabetes among participants was 7 years (IQR = 5–10). The mean (SD) age of the participants was 62.48 (10.32). The participants had a median of 2 prescribed medications (IQR = 2–4), as shown in Table 2.

The number of family members, medication self-efficacy, satisfaction with medication treatments, necessity of medication, concerns about medication, overuse of medication, and harm of medication were all significantly negatively associated with medication burden, with correlation coefficients ranging from −0.38 to −0.11, and all *p* values were < 0.05 (Table 2). Conversely, age, social support, and depression were significantly positively associated with medication burden, with correlation coefficients ranging from 0.15 to 0.43, and all *p* values were < 0.05. In the Medication Beliefs scale, overuse of medication was very strongly correlated with concerns about medication (*r* = 0.82, *p* < 0.01); however, the absolute correlation between overuse of medication and medication burden was lower than that between concerns about medication and medication burden, leading to its exclusion from the linear regression analysis to address multicollinearity.

**Figure 1:**
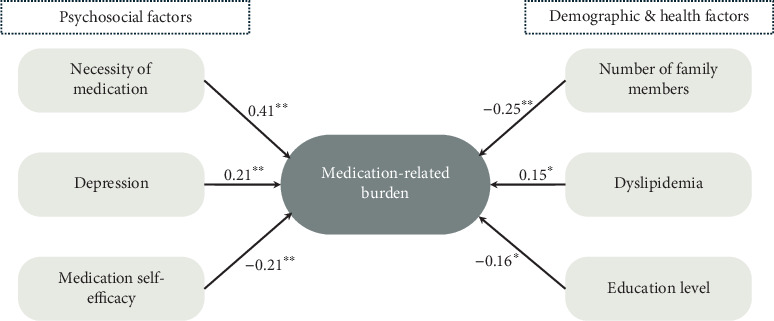
Additional multiple linear regression model of factors associated with medication-related burden (*n* = 261). Note: ⁣^∗^*p* value = 0.002; ⁣^∗∗^*p* < 0.001.

The results of the multiple linear regression analysis revealed that the presence of dyslipidemia (*β* = 0.18, *p* = 0.001) and depression (*β* = 0.16, *p* = 0.005) and increased perceived necessity of medication (*β* = 0.417, *p* < 0.001) were significantly associated with increased medication burden (Table 3). In contrast, higher education (*β* = −0.145, *p* = 0.01), a greater number of family members (*β* = −0.187, *p* = 0.007), and higher self-efficacy in medication use (*β* = −0.133, *p* = 0.035) were significantly associated with a reduction in medication burden. This model explained 48.5% of the variance in medication burden (*F* (14,217) = 14.581, *p* < 0.001). The VIF values for all the variables ranged from 1.12 to 2.64, indicating that there was no multicollinearity. An additional regression model also demonstrated that dyslipidemia, depression, the need for medication, education level, the number of family members, and medication self-efficacy were independently associated with medication burden (see Figure 1). This model explained 44.1% of the variance in medication burden (*F* (6,239) = 31.402, *p* < 0.001). The VIF values for all the variables ranged from 1.07 to 1.39, again indicating no multicollinearity.

## 4. Discussion

Our study indicated that a significant increase in the medication burden was observed in patients with Type 2 diabetes with dyslipidemia, depression, and a greater belief in the necessity of medication use. Conversely, a significant reduction in medication burden was found in patients with higher education levels, a greater number of family members, and greater self-efficacy in medication use.

The participants with higher education levels in this study tended to experience a significantly reduced medication burden. Awad et al. reported significantly lower burden scores related to side effects in individuals with higher educational attainment [31]. Possible explanations include that patients with higher education levels are typically more health conscious and adopt healthier lifestyles [32]. Additionally, they are more aware of disease complications and have better knowledge of self-care, medication adherence, diet management, and greater access to educational resources [33].

While social support can provide motivation and encouragement, it does not necessarily directly reduce the medication burden. Evidence from Yang et al. [21] shows a negligible relationship between medication burden and medication social support, with a correlation coefficient of *r* = 0.11, similar to the findings of the current study (*r* = 0.15). Medication use can negatively impact or disrupt patients' social activities, a common issue that many patients face [17], leading to a perceived burden that social support alone may not alleviate. Interestingly, this study revealed that a greater number of family members was significantly associated with a reduction in medication burden. As the number of family members increases, emotional support and caregiving responsibilities may be shared among more individuals. Family support is considered a crucial factor in facilitating patient recovery [34]. Future studies should explore how family support affects medication burden more than nonfamily support does and assess whether the quality or type of support plays a more important role. Additionally, understanding how cultural factors influence family involvement in caregiving may help explain the significant impact of family support.

Unsurprisingly, the patients with Type 2 diabetes in the current study who also had dyslipidemia experienced a greater medication burden. The presence of multiple conditions can also lead to poorer physical and mental functioning, further diminishing patients' self-management capacity [21]. Many individuals with multiple chronic conditions must adhere to complex self-care and treatment regimens, integrating them smoothly into their daily lives to maintain health [35]. Patients with multiple chronic conditions often face significant treatment burdens, leading to poor health outcomes, reduced adherence to treatment, inefficient resource use, and increased burden on their caregivers [36]. In contrast, while multiple medication use can be burdensome, previous studies have shown that it does not necessarily reduce patient adherence to newly added lipid-lowering therapy [37]. This suggests that physicians should not hesitate to prescribe additional lipid-lowering treatments when clinically necessary. This association requires further investigation in future studies on the Vietnamese population.

The significant role of mental health issues in exacerbating the medication burden has been demonstrated in previous research [21], and similar findings were observed in our study, further reinforcing the association between depression and the medication burden. This connection has important implications for clinical practice, as depression is quite common in Vietnam, with a reported prevalence of 23.2% among patients with diabetes [38]. Evidence indicates that depression is linked to adverse outcomes in individuals with diabetes [39]. Therefore, healthcare professionals should proactively identify patients with diabetes with comorbid depression early to help mitigate their medication burden.

As expected, our study revealed that higher medication self-efficacy significantly reduced the medication burden, which is consistent with prior research [21]. Confidence is a key predictor of an individual's ability to change and maintain health-related behaviors [40]. Patients with higher self-efficacy are more likely to perform tasks effectively, achieve goals, and overcome challenges [41] related to medication use, such as managing side effects, integrating medication regimens into daily life, and maintaining adherence while traveling [21]. This results in better medication adherence. Healthcare professionals should assist patients in enhancing their ability to self-manage medication use to minimize the negative impacts of medication burden on their health and daily lives.

Patients with strong beliefs about the necessity of medications often perceive their illness as chronic and experience more symptoms [42]. Our study revealed that this belief may be independently associated with increased medication burden, a relationship not identified in previous research [21]. This could be explained by the fact that while patients recognize the importance of medications for managing their condition, in more severe cases, where multiple medications are needed, they may experience a greater burden from treatment adherence, including psychological stress and the complexity of medication management. However, the perception of the necessity of medications can vary depending on individual circumstances and the specific illness. Thus, further research is needed to better understand how this belief impacts medication burden and to find strategies that balance treatment adherence with reducing patient burden.

Although the study provides valuable insights that can form the basis for interventions aimed at reducing medication burden, these interventions need to be adapted to the local context of different regions or countries. Future research should focus on cross-cultural studies to explore how these factors interact in different regional contexts.

### 4.1. Limitations

This study has several limitations. First, the data were collected through self-report questionnaires from patients, which may introduce subjectivity and response bias. Second, the study sample was limited to a single locality, which may affect the generalizability of the results to the broader population of patients with Type 2 diabetes in Vietnam and other countries. Additionally, other psychosocial factors, such as health literacy, financial stress, fear of hypoglycemia, and low mood, that could influence medication burden were not fully explored in this study. Future research should expand the geographical scope and include these psychosocial factors to gain a more comprehensive understanding of the medication burden among patients with Type 2 diabetes.

## 5. Conclusions

This study demonstrated that factors such as the presence of dyslipidemia, depression, and a strong belief in the necessity of medication were significantly associated with an increased medication burden in patients with Type 2 diabetes, whereas higher education, a greater number of family members, and greater self-efficacy in medication use played a role in reducing the medication burden. These findings highlight the importance of considering psychosocial factors in managing medication burden. Furthermore, developing interventions to alleviate the medication burden should be a key focus of future research to improve the quality of life of patients with Type 2 diabetes.

## Figures and Tables

**Table 1:** Participants' demographic and health characteristics (*n* = 261).

**Characteristics**		**n**	**%**	**Medication burden median (IQR)**	**p** ** value**
*Demographics*					
Age group (year)	< 60	95	36.4	5 (2–6)	
	≥ 60	166	63.6	5 (4–8)	**< 0.001**
Gender	Women	167	64.0	5 (4–8)	**< 0.001**
	Men	94	36.0	4 (2–5)	
Education level	Lower secondary or below	108	42.7	6 (5–9)	**< 0.001**
	Upper secondary	74	29.2	5 (4–6)	
	College or intermediate	33	13.0	4 (2–7)	
	University or higher	38	15.0	2 (2–5)	
Marital status	Single or widowed	68	27.4	7 (5–11)	**< 0.001**
	Married	180	72.6	5 (2–6)	
Occupation status	Homemaker	66	25.5	5 (3–6)	
	Retired	93	35.9	6 (5–8)	**< 0.001**
	Farmer	44	17.0	5 (4–6)	
	Employed	56	21.6	3 (2–5.5)	

*Health characteristics*					
Hypertension	No	28	11.0	3.5 (2–6)	
	Yes	226	89.0	5 (3–7)	0.120
Dyslipidemia	No	170	66.9	5 (2–6)	
	Yes	84	33.1	6 (3.5–10.5)	**< 0.001**
Arthritis	No	183	72.0	5 (2–6)	
	Yes	71	28.0	5 (4–12)	**0.002**
Angina	No	205	80.7	5 (2–7)	
	Yes	49	19.3	6 (5–8)	**0.001**
Osteoporosis	No	213	83.9	5 (2–6)	
	Yes	41	16.1	10 (4–15)	**< 0.001**

*Note:* Bold numbers indicate statistical significance.

Abbreviation: IQR, interquartile range.

**Table 2:** Spearman's rho correlation analysis of demographic and health characteristics, psychosocial factors, and medication burden (*n* = 261).

**Variables**		**Median (IQR)**	**Spearman's rho correlation**												
			**1**	**2**	**3**	**4**	**5**	**6**	**7**	**8**	**9**	**10**	**11**	**12**	**13**
1	Age, mean (SD)	62.48 (10.32)	1												
2	Number of family members	4 (3–6)	−0.16⁣^∗∗^	1											
3	Number of medications in prescription	2 (2–4)	−0.06	0.66⁣^∗∗^	1										
4	Duration of diabetes (year)	7 (5–10)	0.29⁣^∗∗^	0.20⁣^∗∗^	0.22⁣^∗∗^	1									
5	Medication self-efficacy	23 (20–24)	−0.36⁣^∗∗^	0.29⁣^∗∗^	0.25⁣^∗∗^	−0.05	1								
6	Medication social support	3.63 (3.25–3.88)	0.14⁣^∗^	0.35⁣^∗∗^	0.44⁣^∗∗^	0.20⁣^∗∗^	0.16⁣^∗∗^	1							
7	Satisfaction with medication treatments	83.33 (72.22–94.44)	−0.24⁣^∗∗^	−0.12	−0.17⁣^∗∗^	−0.24⁣^∗∗^	0.34⁣^∗∗^	−0.01	1						
8	Depression	1 (0–2)	0.21⁣^∗∗^	−0.08	0.01	−0.01	−0.23⁣^∗∗^	0.15⁣^∗^	−0.07	1					
9	Necessity of medication	9 (8–13)	0.03	−0.48⁣^∗∗^	−0.63⁣^∗∗^	−0.20⁣^∗∗^	−0.12	−0.49⁣^∗∗^	0.23⁣^∗∗^	−0.21⁣^∗∗^	1				
10	Concerns about medication	18 (12–22)	−0.27⁣^∗∗^	0.63⁣^∗∗^	0.66⁣^∗∗^	0.23⁣^∗∗^	0.53⁣^∗∗^	0.45⁣^∗∗^	0.10	−0.21⁣^∗∗^	−0.51⁣^∗∗^	1			
11	Overuse of medication	8 (6–13)	−0.14⁣^∗^	0.64⁣^∗∗^	0.69⁣^∗∗^	0.20⁣^∗∗^	0.44⁣^∗∗^	0.46⁣^∗∗^	0.00	−0.12⁣^∗^	−0.53⁣^∗∗^	0.82⁣^∗∗^	1		
12	Harm of medication	13 (11–14)	−0.12	0.34⁣^∗∗^	0.47⁣^∗∗^	0.14⁣^∗^	0.24⁣^∗∗^	0.31⁣^∗∗^	0.12	0.04	−0.42⁣^∗∗^	0.63⁣^∗∗^	0.53⁣^∗∗^	1	
13	Medication burden	5 (3–7)	0.28⁣^∗∗^	−0.15⁣^∗^	0.00	0.10	−0.38⁣^∗∗^	0.15⁣^∗^	−0.30⁣^∗∗^	0.43⁣^∗∗^	−0.31⁣^∗∗^	−0.27⁣^∗∗^	−0.20⁣^∗∗^	−0.11	1

Abbreviations: IQR, interquartile range; SD, standard deviation.

⁣^∗^Correlation is significant at the 0.05 level (2-tailed).

⁣^∗∗^Correlation is significant at the 0.01 level (2-tailed).

**Table 3:** Univariate and multiple linear regression analyses of factors associated with medication burden (*n* = 261).

**Variables**	**Univariate linear regression**				**Multiple linear regression**			
	**B**	**S** **E**	**β**	**p** ** value**	**B**	**S** **E**	**β**	**p** ** value**
Necessity of medication	0.427	0.071	0.351	**< 0.001**	0.464	0.066	0.417	**< 0.001**
Number of family members	−0.224	0.047	−0.281	**< 0.001**	−0.135	0.050	−0.187	**0.007**
Dyslipidemia (ref. no)	0.507	0.151	0.207	**0.001**	0.413	0.118	0.180	**0.001**
Depression	0.246	0.033	0.420	**< 0.001**	0.088	0.031	0.160	**0.005**
Education level (ref. lower secondary or below)	−0.357	0.062	−0.34	**< 0.001**	−0.151	0.058	−0.145	**0.010**
Medication self-efficacy	−0.334	0.065	−0.304	**< 0.001**	−0.132	0.062	−0.133	**0.035**
Harm of medication	0.110	0.047	0.144	**0.020**	0.080	0.042	0.114	0.058
Marital status (ref. single or widowed)	−0.969	0.145	−0.392	**< 0.001**	−0.238	0.165	−0.102	0.150
Sex (ref. women)	−0.649	0.144	−0.270	**< 0.001**	−0.192	0.150	−0.086	0.203
Concerns about medication	0.213	0.058	0.221	**< 0.001**	0.055	0.070	0.062	0.434
Medication social support	0.193	0.071	0.167	**0.007**	0.045	0.065	0.042	0.495
Occupation status (ref. homemaker or retired)	−0.364	0.144	−0.156	**0.012**	0.093	0.149	0.042	0.535
Satisfaction with medication treatments	−0.308	0.076	−0.244	**< 0.001**	−0.042	0.069	−0.036	0.546
Age	0.028	0.007	0.247	**< 0.001**	−0.001	0.006	−0.007	0.915
Duration of diabetes (year)	0.126	0.067	0.117	0.060				
Number of medications in prescription	0.070	0.043	0.102	0.102				
Hypertension (ref. no)	0.277	0.231	0.075	0.233				

*Note:* B—unstandardized coefficient; *β*—standardized coefficient; bold numbers indicate statistical significance.

Abbreviation: SE, standard error.

## Data Availability

The data that support the findings of this study are available from the corresponding author (i.e., upon reasonable request).

## References

[1] Hossain M. J., Al-Mamun M., Islam M. R. (2024). Diabetes Mellitus, the Fastest Growing Global Public Health Concern: Early Detection Should Be Focused. *Health Sci Rep*.

[2] International Diabetes Federation (2021). Facts & Figures. https://idf.org/about-diabetes/diabetes-facts-figures/.

[3] Insights10 (2023). Vietnam Diabetes Devices Market Analysis. https://www.insights10.com/report/vietnam-diabetes-devices-market-analysis/?srsltid=AfmBOooFhIDsKdpdJjaabbjGsbOO-xpUP4zPk2MdBFxDWVaTKDTIkljN.

[4] Viet Nam Social Security (2024). Effective Cost Management of Diabetes. https://vss.gov.vn/english/news/Pages/vietnam-social-security.aspx?CateID=198%26ItemID=11942.

[5] World Health Organization Diabetes in Viet Nam. https://www.who.int/vietnam/health-topics/diabetes.

[6] Hoa N. T., Derese A., Peersman W., Markuns J. F., Willems S., Tam N. M. (2020). Primary Care Quality in Vietnam: Perceptions and Opinions of Primary Care Physicians in Commune Health Centers – A Mixed-Methods Study. *PLoS One*.

[7] Le Ho Thi Q.-A., Pype P., Wens J. (2024). Continuity of Primary Care for Type 2 Diabetes and Hypertension and Its Association With Health Outcomes and Disease Control: Insights From Central Vietnam. *BMC Public Health*.

[8] Duong D. B., Van M. H., Ngo L. H., Ellner A. L. (2019). Readiness, Availability and Utilization of Rural Vietnamese Health Facilities for Community Based Primary Care of Non-Communicable Diseases: A CrossSectional Survey of 3 Provinces in Northern Vietnam. *International Journal of Health Policy and Management*.

[9] Nguyen H. T. T., Moir M., Nguyen T. X. (2018). Health-Related Quality of Life in Elderly Diabetic Outpatients in Vietnam. *Patient Preference and Adherence*.

[10] Nguyen V. Q., Tran T. A., Do T. K. H. (2024). The Status of Treatment Adherence of Type 2 Diabetes Patients at Hanoi Heart Hospital, 2023. *Vietnam Journal of Community Medicine*.

[11] Huong D. T. L., Hang N. T., Ly N. K. (2023). Determination of Drug-Related Problems Among Type 2 Diabetes Outpatients in a Hospital in Vietnam: A Cross-Sectional Study. *PLoS One*.

[12] Jamal Noori A., Jabbar Kadhim D., Abdulhasan Al-Hilal M. (2022). Medication-Related Burden Among Patients With Diabetes Mellitus and its Relation to Diabetic Control Parameters: An Observational Study. *F1000Res*.

[13] Saydah S. H. (2018). *Medication Use and Self-Care Practices in Persons With Diabetes*.

[14] İnci H. (2021). Evaluation of Multiple Drug Use in Patients With Type 2 Diabetes Mellitus. *Diabetology International*.

[15] Jyrkkä J., Enlund H., Korhonen M. J., Sulkava R., Hartikainen S. (2009). Polypharmacy Status as an Indicator of Mortality in an Elderly Population. *Drugs & Aging*.

[16] Bekalu A. F., Yenit M. K., Tekile M. T., Birarra M. K. (2022). Medication-Related Burden and Associated Factors Among Diabetes Mellitus Patients at Felege Hiwot Comprehensive Specialized Hospital in Northwest Ethiopia. *Frontiers in Clinical Diabetes and Healthcare*.

[17] Mohammed M. A., Moles R. J., Chen T. F. (2016). Medication-Related Burden and Patients’ Lived Experience With Medicine: A Systematic Review and Metasynthesis of Qualitative Studies. *BMJ Open*.

[18] Baah-Nyarkoh E., Alhassan Y., Dwomoh A. K., Kretchy I. A. (2023). Medicated-Related Burden and Adherence in Patients With Co-Morbid Type 2 Diabetes Mellitus and Hypertension. *Heliyon*.

[19] Mehta M., Kapoor S. (2018). Role of Psychosocial Factors in the Management of Health Problems. *Psychosocial Interventions for Health and Well-Being*.

[20] Cross S. P., Karin E., Staples L. G. (2022). Factors Associated With Treatment Uptake, Completion, and Subsequent Symptom Improvement in a National Digital Mental Health Service. *Internet Interventions*.

[21] Yang C., Zhu S., Hui Z., Mo Y. (2023). Psychosocial Factors Associated With Medication Burden Among Community-Dwelling Older People With Multimorbidity. *BMC Geriatrics*.

[22] Risser J., Jacobson T. A., Kripalani S. (2007). Development and Psychometric Evaluation of the Self-Efficacy for Appropriate Medication Use Scale (SEAMS) in Low-Literacy Patients With Chronic Disease. *Journal of Nursing Measurement*.

[23] Lehavot K., Huh D., Walters K. L., King K. M., Andrasik M. P., Simoni J. M. (2011). Buffering Effects of General and Medication-Specific Social Support on the Association Between Substance Use and HIV Medication Adherence. *AIDS Patient Care and STDs*.

[24] Bharmal M., Payne K., Atkinson M. J., Desrosiers M. P., Morisky D. E., Gemmen E. (2009). Validation of an Abbreviated Treatment Satisfaction Questionnaire for Medication (TSQM-9) Among Patients on Antihypertensive Medications. *Health and Quality of Life Outcomes*.

[25] Tran V., Nguyen T. K., Dewey R. S., Le M. H., Pham D. T. (2023). Depression, Anxiety, and Psychological Distress in Vietnamese Pharmacy and Non-Pharmacy Students During COVID-19 Pandemic. *JACCP: Journal of the American College of Clinical Pharmacy*.

[26] Nguyen T., Cao H. T. K., Quach D. N. (2019). The Vietnamese Version of the Brief Illness Perception Questionnaire and the Beliefs About Medicines Questionnaire: Translation and Cross-Cultural Adaptation. *Tropical Medicine & International Health*.

[27] Tran V.-T., Harrington M., Montori V. M., Barnes C., Wicks P., Ravaud P. (2014). Adaptation and Validation of the Treatment Burden Questionnaire (TBQ) in English Using an Internet Platform. *BMC Medicine*.

[28] Walde P., Völlm B. A. (2003). The TRAPD Approach as a Method for Questionnaire Translation. *Frontiers in Psychiatry*.

[29] Chan Y. H. (2003). Biostatistics 104: Correlational Analysis. *Singapore Medical Journal*.

[30] Templeton G. F. (2011). A Two-Step Approach for Transforming Continuous Variables to Normal: Implications and Recommendations for IS Research. *Communications of the Association for Information Systems*.

[31] Awad A., Alhadab A., Albassam A. (2020). Medication-Related Burden and Medication Adherence Among Geriatric Patients in Kuwait: A Cross-Sectional Study. *Frontiers in Pharmacology*.

[32] Zheng X., Xiao F., Li R. (2019). The Effectiveness of Hypertension Management in China: A Community-Based Intervention Study. *Primary Health Care Research & Development*.

[33] Persell S. D., Keating N. L., Landrum M. B. (2004). Relationship of Diabetes-Specific Knowledge to Self-Management Activities, Ambulatory Preventive Care, and Metabolic Outcomes. *Preventive Medicine*.

[34] Liu L., Huang W., Huang Z. (2022). Relationship Between Family Caregiver Burden and Medication Adherence in Patients With Mechanical Valve Replacement: A Structural Equation Model. *Patient Preference and Adherence*.

[35] Eton D. T., Anderson R. T., St Sauver J. L., Rogers E. A., Linzer M., Lee M. K. (2022). Longitudinal Trajectories of Treatment Burden: A Prospective Survey Study of Adults Living With Multiple Chronic Conditions in the Midwestern United States. *Journal of Multimorbidity and Comorbidity*.

[36] Sav A., King M. A., Whitty J. A. (2015). Burden of Treatment for Chronic Illness: A Concept Analysis and Review of the Literature. *Health Expectations*.

[37] Robertson T. A., Cooke C. E., Wang J., Shaya F. T., Lee H. Y. (2008). Effect of Medication Burden on Persistent Use of Lipid-Lowering Drugs Among Patients With Hypertension. *The American Journal of Managed Care*.

[38] Tran N. M. H., Nguyen Q. N. L., Vo T. H., Le T. T. A., Ngo N. H. (2021). Depression Among Patients With Type 2 Diabetes Mellitus: Prevalence and Associated Factors in Hue City, Vietnam. *Diabetes, Metabolic Syndrome and Obesity*.

[39] Katon W. J., Rutter C., Simon G. (2005). The Association of Comorbid Depression With Mortality in Patients With Type 2 Diabetes. *Diabetes Care*.

[40] Rieder A., Eseryel U. Y., Lehrer C., Jung R. (2021). Why Users Comply With Wearables: The Role of Contextual Self-Efficacy in Behavioral Change. *International Journal of Human Computer Interaction*.

[41] Huang L., Li L., Zhang Y., Li H., Li X., Wang H. (2013). Self-Efficacy, Medication Adherence, and Quality of Life Among People Living With HIV in Hunan Province of China: A Questionnaire Survey. *The Journal of the Association of Nurses in AIDS Care*.

[42] Ismail H., Wan Azmi W. U. H., Loganathan M., Usir E. (2021). Adherence Level and Medication Beliefs Among Care Homes Residents in Klang Valley. *International Journal of Pharmaceuticals, Nutraceuticals and Cosmetic Science (IJPNaCS)*.

